# Therapy-resistant dry itchy eyes

**DOI:** 10.1186/s12348-019-0178-7

**Published:** 2019-07-23

**Authors:** Rima Wardeh, Volker Besgen, Walter Sekundo

**Affiliations:** University Eye Clinic of Marburg, UKGM – Philipps-University Marburg, Maburg, Germany

## Abstract

An 8 years old male presented to our clinic with dry eye symptomes. Different therapiy attemps were made in the last few months and did not lead to any improvement. Examining this patient revealed multiple signs of vitamin A deficiency, which could confirmed by laboratory examination. The initial substitution of vitamin A led to a fast rehabilitation and a following nutrition consulting kept the patient symptom-free over 6 month follow up. Vitamin A deficiency -although rare in the developed countries- is an importent differential diagnosis of the dry eye especially in children. Vitamin A deficiency not only causes ocular manifistaion, but also general symptoms. Dietary change and initial subtitution is the key element for a fast and sustaining improvement.

## Medical history

An 8-year-old male child was referred to our pediatric ophthalmology department because of burning sensation and itching in both eyes during the last 4 months. His mother reported that the child was always pinching his eyes while reading or focusing. Topical therapy with dexamethasone eye drops, antihistamine eye drops (ketotifen), antibiotic eye drops (ofloxacine) and immunosuppressive eye drops (cyclosporine 1%) led to no improvement. The patient was receiving treatment for neurodermatitis from his pediatrician. Several allergies were known. Furthermore, the child was suffering from recurrent urinary tract infection, diarrhea, bronchiti, and otitis media in the last months. He had to be treated with systemic antibiotics multiple times due to these problems. The child’s parents immigrated from the Philippines, but have been living in Germany for many years.

The best corrected distance visual acuity was 6/15 (Snellen’s chart) in the right eye and 6/12 in the left eye. Slit lamp examination showed remarkable dryness of the cornea with superficial punctate keratitis (SPK) of both eyes. The conjunctiva was not injected or irritated but had foamy secretions. Eversion of the upper lid showed a lot of follicles on the tarsal conjunctiva. With these findings, the diagnosis of immunogenic conjunctivitis due to neurodermatitis was made. The patient was asked to stop all the eye drops and to apply only preservative-free artificial tears regularly. Four weeks later, the follow-up examination showed no improvement in the visual acuity nor in the corneal surface. In addition, the conjunctiva had developed triangular-shaped superficial spots with keratinization in the bulbar conjunctiva nasally, inferiorly, and temporally near the limbus of both eyes. With these findings, the diagnosis of conjunctival and corneal xerosis due to vitamin A deficiency was suspected. Laboratory findings showed vitamin A levels of 20.0 μg/l (normal value 260–490 μg/l). Hence, we referred the patient to the pediatric department in order to substitute vitamin A and to exclude any related malabsorption or other diseases.

The general physical examination of the child showed no pathological findings. All laboratory findings were also within normal limits, except for vitamin D deficiency (25-hydroxyvitamin D 6.5 μg/l, normal 20–50 μg/l).

Following the laboratory tests, the parents were asked about the nutritional habits of the child. His food preference included white bread, rice, pasta, sausage, and chocolate. He did not consume any fruits or vegetables.

The diagnosis of vitamin A and vitamin D deficiency due to malnutrition was made. The child was treated with oral retinol (vitamin A1 or axerophthol) 100,000 units/day and vitamin D 1000 units/day. In addition, he was referred to a nutrition specialist for counseling.

**Fig. 1:**
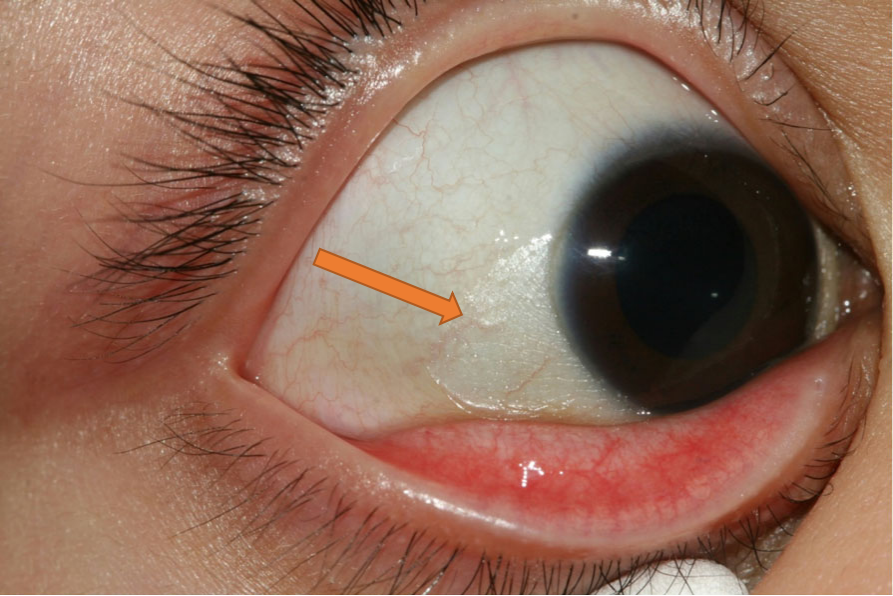
Bitot’s spots and keratinization of the nasal, inferior, and temporal conjunctiva (see arrows)

**Fig. 2:**
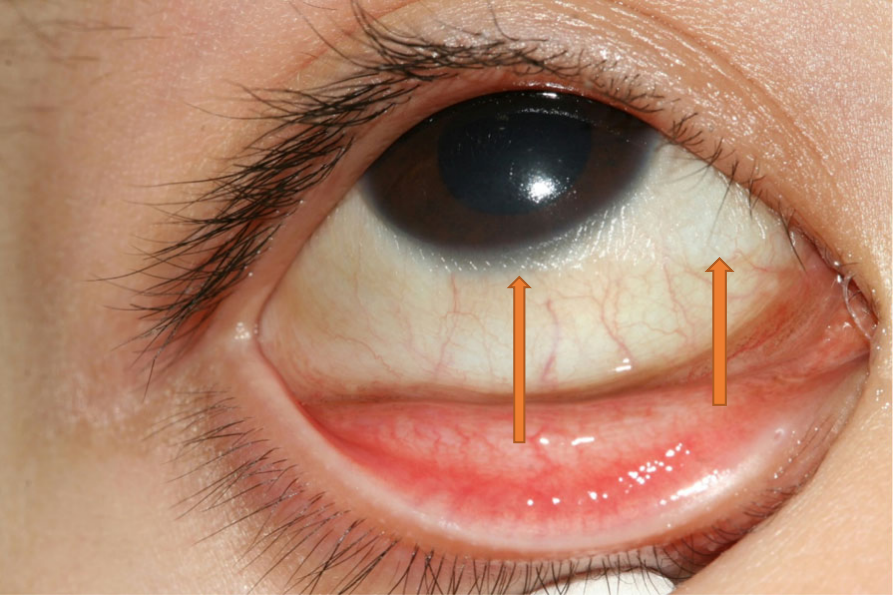
Bitot’s spots and keratinization of the nasal, inferior, and temporal conjunctiva (see arrows)

**Fig. 3:**
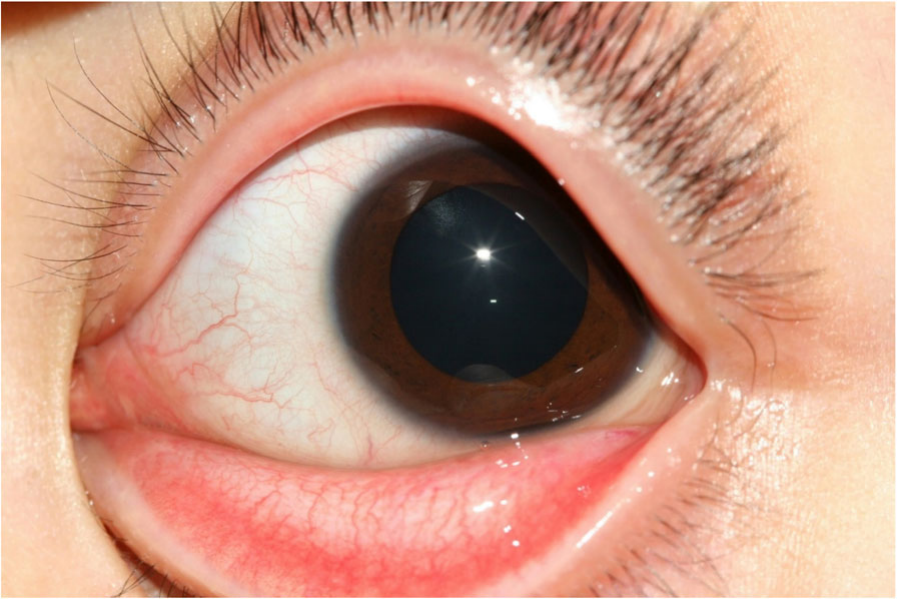
The conjunctiva and the cornea after treatment

Four weeks following therapy, the child’s complaints decreased. The visual acuity rose to 6/7.5 (Snellen’s chart) in both eyes and the slit lamp examination showed unremarkable conjunctiva and clear cornea with minimal superficial punctate keratitis (SPK). The general situation of the patient was also better, which caused a lower frequency of urinary tract infection, diarrhea, bronchitis, and otitis media. Follow-up laboratory tests showed vitamin A level within normal limits of 267 μg/l. The patient continued the substitutional therapy with retinol and vitamin D for 3 months. Our last follow-up, 3 months after discontinuing the substitutional therapy, showed an asymptomatic patient with a normal eye examination (Figs. 1, 2 and 3).

## Discussion

Vitamin A deficiency (VAD) is an important nutritional problem worldwide. In 2009, the World Health Organization (WHO) estimated that about 127 million children in preschool age in the developing world suffered from vitamin A deficiency, which is defined as retinol serum concentration < 0.70 μmol/l. Xerophthalmia exists in 4.4 million of these children [10] (Table 1).

**Table 1:** The World Health Organization (WHO) classification of xerophthalmia in 1995 [7]

WHO’s classification of xerophthalmia [7]	
XN	Night blindness
X1A	Conjunctival xerosis
X1b	Bitot’s spots
X2	Corneal xerosis
X3A	Corneal ulceration/keratomalacia < 1/3 corneal surface
X3B	Corneal ulceration/keratomalacia ≥ 1/3 corneal surface
XS	Corneal scar
XF	Xerophthalmic fundus

The prevalence of vitamin A deficiency differs worldwide. WHO reported that about half of the children affected with VAD reside in south and south-east Asia [10]. Since VAD is very rare in Germany, there is no data about its prevalence there. In addition to geographic factors, many other risk factors for VAD have been identified through epidemiological studies. Preschool age children are at higher risk for VAD. This could be because of the higher requirements for growth and the restricted body storage of vitamin A at this young age [6]. The educational status of the parents and the economical environment are also very important, since they can affect accessing a good balanced diet [6]. Nutritional habits related to cultural behaviors can also play a role in VAD [5].

Vitamin A is one of the lipid-soluble vitamins that is stored in the liver and cannot be produced in human bodies. Vitamin A has to be supplied through diet and can be found in leafy greens, fruits, carrots, oranges, and animal products such as milk, eggs, and liver [3]. Causes of VAD include insufficient uptake from diet, alcohol consumption, pancreatitis, malabsorption, and biliary obstruction.

It is important to point out that VAD is associated with increased infections, such as measles and bronchitis, as well as diarrhea. That is why VAD is combined with increased mortality in children [3].

Vitamin A plays a very important role in the visual process. In addition to its role in the synthesis of retinal rhodopsin, it is essential in the differentiation of the stratified squamous epithelium of the eye surface. The ocular manifestation of VAD is called xerophthalmia, which includes night blindness (nyctalopia), conjunctival xerosis, and corneal xeropthalmia [2, 8]. The most common symptoms of xerophthalmia include blurred vision, dryness of the eyes, and night blindness.

Conjunctival xerosis manifests as dryness of the conjunctiva with typical Bitot’s spots. They were first described by the French physician Charles Bitot in 1863 [4]. Bitot’s spots present as white elevated lesions on the bulbar conjunctiva near the limbus*,* covered with a foamy cheesy material usually starting in the temporal side of the limbus. In more severe cases, all quadrants can be involved [8]. Histological changes include loss of the goblet cells and keratinizing metaplasia of the outer layers of the conjunctiva. The material covering Bitot’s spots contain frayed keratin as well as gram-positive bacilli called “xerosis bacillus” [8]. Vital staining is an important diagnostic tool in the evaluation of the ocular surface changes. Staining with lissamine green can be very helpful in detecting the early corneal changes and may be superior to fluorescent staining in these cases [1].

In more advanced cases of VAD like in the case present here, the cornea develops superficial punctate keratopathy (SPK), usually starting in the inferonasal quadrants and involving the whole cornea as the deficiency gets more severe. Untreated cases may cause corneal ulceration and keratomalacia, which may result in corneal scars and blindness [8].

It is thought that these SPK could be caused by a toxic effect of the material covering Bitot’s spots as well as the loss of goblet cells, which causes a reduction of the mucous layer of the tear film [8].

Xerophthalmia is a medical emergency requiring immediate treatment with vitamin A to save the eye as well as the life of the child [11]. It usually responds quickly to vitamin A therapy. The night blindness usually resolves 1–2 days after treatment. Bitot’s spots need several days to a few weeks to resolve, whereas the corneal lesions start to heal within 1 week [7, 9].

The WHO has determined the therapeutic doses of vitamin A supplementation depending on the age of the child. The first dose should be given orally; 50,000 IU for children younger than 6 months, 100,000 IU between 6–12 months and 200,000 for children older than 12 months. The same doses should be repeated the next day and 2 weeks later. Local therapy of artificial eye drops and antibiotic eye drops or ointments may be useful [11]. Nutritional consulting and regular follow-ups are necessary to prevent and detect any signs of recurrence.

## Conclusion

- Inappropriate diet is an important cause of vitamin A deficiency beside alcohol consumption, pancreatitis, malabsorption, and biliary obstruction.

- Chronic vitamin A deficiency as a systemic syndrome may convert to a disease with recurring infections beside ocular manifestations.

- Although vitamin A deficiency is very rare in developed societies, it should be part of the differential diagnosis of dry eye syndrome in children.

- The suitable substitution is essential and causes a rapid therapeutic effect.

- Nutritional consulting, including changing unsuitable nutrition habits, is one key element in treating and preventing the recurrence of this disease.

## Data Availability

Data sharing is not applicable to this article as no datasets were generated or analyzed during the current study.
